# Mechanisms of Resistance to Immunotherapy in Hepatocellular Carcinoma

**DOI:** 10.2147/JHC.S291553

**Published:** 2023-11-03

**Authors:** Giulia Francesca Manfredi, Ciro Celsa, Chloe John, Charlotte Jones, Nicole Acuti, Bernhard Scheiner, Claudia Angela Maria Fulgenzi, James Korolewicz, Matthias Pinter, Alessandra Gennari, Francesco A Mauri, Mario Pirisi, Rosalba Minisini, Federica Vincenzi, Michela Burlone, Cristina Rigamonti, Matteo Donadon, Giuseppe Cabibbo, Antonio D’Alessio, David James Pinato

**Affiliations:** 1 Department of Surgery & Cancer, Imperial College London, Hammersmith Hospital, London, UK; 2 Department of Translational Medicine, Università Del Piemonte Orientale, Novara, Italy; 3 Section of Gastroenterology & Hepatology, Department of Health Promotion, Mother and Child Care, Internal Medicine and Medical Specialties, PROMISE, University of Palermo, Palermo, Italy; 4 Department of Surgical, Oncological and Oral Sciences (Di.chir.on.s.), University of Palermo, Palermo, Italy; 5 Division of Gastroenterology and Hepatology, Department of Internal Medicine III, Medical University of Vienna, Vienna, Austria; 6 Department of Medical Oncology, University Campus Bio-Medico of Rome, Rome, Italy; 7 Division of Oncology, Department of Translational Medicine, University of Piemonte Orientale, Novara, Italy; 8 Division of Internal Medicine, AOU Maggiore della Carità, Novara, Italy; 9 Department of Health Science, Università Del Piemonte Orientale, Novara, Italy; 10 Department of Surgery, University Maggiore Hospital della Carità, Novara, Italy

**Keywords:** immune checkpoint inhibitors, liver cancer, recurrence, tumor microenvironment

## Abstract

Systemic treatment for advanced hepatocellular carcinoma (HCC) has been revolutionized over the last few years following the approval of immune checkpoint inhibitors (ICI). Despite the promising survival extension seen with ICI combination regimens, responses are not universally seen and the optimal partner for programmed cell death 1 pathway inhibitors remains to be identified. Even fewer encouraging results have been demonstrated with ICI used for monotherapy. Several mechanisms of resistance have been described so far, involving characteristics of cancer cells (intrinsic mechanisms) and of the surrounding tumor microenvironment (extrinsic mechanisms). Factors related to therapy may also contribute to the development of resistance. Increasing research efforts are being dedicated to the discovery of novel approaches and targets to overcome resistance, some of which may be introduced into clinic in the future. Herein we describe a selection of resistance mechanisms that have been involved in impairing response to ICI and propose potential therapeutic approaches to overcome resistance.

## Introduction

Hepatocellular carcinoma (HCC) represents the most common primary liver cancer and a globally burdensome oncological diagnosis, ranking as the sixth most frequently diagnosed cancer and the third most common cause of cancer death worldwide.^1^ Unlike other malignancies, both incidence and mortality from HCC are expected to rise by more than 55% in the next few decades.^2^ HCC usually develops in the context of chronic liver disease, with Hepatitis B virus (HBV) and Hepatitis C virus (HCV) infection and alcohol abuse representing the most prevalent etiologies worldwide. However, the proportion of HCC cases arising in the setting of underlying metabolic dysfunction-associated fatty liver disease (MAFLD) is rapidly increasing, and it is expected that MALFD will become the leading risk factor for HCC in the years to come.^3^ Improved management of underlying chronic liver disease is likely to reduce the risk of cirrhosis decompensation and the ensuing mortality, leading to an increase in the number of patients with prolonged survival from cirrhosis but still at risk for developing HCC. On the other hand, the increasing prevalence of MAFLD, associated with the lack of effective surveillance strategies in patients with non-cirrhotic MAFLD and with the intrinsic difficulty of ultrasound surveillance in cirrhotic patients, is likely related to the persistently high number of cases diagnosed at advanced stages.^4^ For these patients, systemic therapy still remains the standard of care.

The advent of novel, more effective systemic therapies has markedly revolutionized the prognostic outlook of patients with advanced HCC. Monotherapies with the anti-programmed cell death protein-1 (PD-1) targeting agents nivolumab and pembrolizumab received accelerated approval by the Food and Drug Administration (FDA), based on unexpectedly high radiological response rates observed in uncontrolled early-phase clinical trials.^5^,^6^ However, PD-1 monotherapy failed in showing a significant overall survival (OS) benefit in Phase III RCTs and only pembrolizumab was granted full FDA approval.^7^,^8^ Primary disease progression on nivolumab monotherapy was reported in 37% of patients,^7^ suggesting that resistance to this type of therapeutic approach is common. Follow-up studies of tislelizumab and durvalumab, two monoclonal antibodies directed against PD-1 and programmed cell death ligand-1 (PD-L1), respectively, demonstrated the non-inferiority of PD-1/PD-L1 blockade against sorafenib, with meta analyses showing improved therapeutic index over tyrosine kinase inhibitors (TKIs).^9^

Conversely, it is now known that ICI-based combination regimens provide a significant improvement in OS compared to sorafenib in phase III RCTs, shifting the expected survival of patients with advanced disease from approximately 6 months to up to 20 months.^10^,^11^ The combinations of atezolizumab (anti-PD-L1) plus bevacizumab (anti-vascular endothelial growth factor [VEGF]), and durvalumab (anti-PD-L1) plus another ICI (tremelimumab, an anti-Cytotoxic T-Lymphocyte Antigen 4 [CTLA-4]) have obtained full FDA approval for first-line treatment, whereas the combination of nivolumab plus anti-CTLA-4 ipilimumab has been granted accelerated approval for patients who have been previously treated with sorafenib.^12^ The rationale behind combining ICI with anti-VEGF is that VEGF inhibition may regulate and suppress elements of the tumor immune microenvironment (TiME), including regulatory T cells (T-regs), myeloid-derived suppressor cells (MDSCs), and tumor-associated macrophages (TAM). On the other hand, the combined blockade of CTLA-4 and PD-1 in ICI doublets has a distinct and non-redundant immune-biologic role within the cancer immunity cycle. CTLA-4 inhibition drives immune-suppression in tumor-antigen presenting cells and T-regs, while PD-1/PD-L1 blockade predominantly downregulates the effectiveness of the cytotoxic CD8+ T cell response.^13^ Unfortunately, combinations of ICI plus TKI, such as atezolizumab plus cabozantinib or pembrolizumab plus lenvatinib, did not statistically improve OS compared to TKI monotherapy in phase III RCTs.^14^,^15^ Overall, compared to ICI monotherapies, ICI-based doublets are associated with improved outcomes, and this is probably related to the added benefit of each component. However, the proportion of patients obtaining long-term survival benefit still remains limited and does not exceed 20–30%. This has led to the development of triplet regimens that have been investigated in phase I/II trials (such as nivolumab plus ipilimumab and cabozantinib) or are currently under investigation (such as atezolizumab plus bevacizumab and ipilimumab).^16^,^17^

HCC is characterized by high clinical and biological heterogeneity, translating into an extremely variable prognosis.^18^ The diagnosis of HCC can be performed by using validated imaging criteria, without the need for histological confirmation in patients with cirrhosis.^19^ This has inevitably led to a relatively limited understanding of the molecular mechanisms associated with prognosis and treatment response compared to other solid tumors. Nevertheless, it is well known that HCC is characterized by a high transcriptional and genetic heterogeneity.^20^ Research on molecular signatures, which could potentially predict outcomes, led to the identification of two major subclasses of HCC: one characterized by intrinsic cell proliferation, and the other defined as the non-proliferative class. The former is denoted by poor tumor cell differentiation and associated with chromosomal instability, as well as p53 tumor suppressor gene (TP53) mutations. Potentially actionable driver linked to this class is transforming growth factor beta (TGF-beta) signaling. The non-proliferative class is associated with more differentiated HCC, presence of mutations in the Wnt/beta-catenin pathway and with a better prognosis.^21^ Utilization of high throughput sequencing technologies led to a further deepening in the understanding of the functional characteristics recapitulated within the HCC TiME. First, the presence of a percentage of HCC with expression of markers of inflammatory response, named as immune class, was identified.^22^ A subsequent study improved the classification.^23^ Three phenotypic classes were defined: an immune class, characterized by high immune cell infiltration and PD-1/PD-L1 pathway activation, an immune intermediate class, and an immune-excluded class, which conversely is associated with low immune cell infiltration, low expression of PD-1 and PD-L1 and high frequency of chromosomal aberrations.^23^ A fourth subclass, defined as immune-desert pattern, has also been described; it is distinguished by an impoverishment of the immune component leading to immune ignorance and a lack of priming T cell.^24^

While useful in understanding the pathogenesis of HCC, implementation of these classifications in clinical practice remains limited, and prospective studies proving their predictive performance are lacking. In addition to molecular characteristics, the heterogeneity of HCC etiology has become the subject of debate with regard to its differential ability to lead to effective immune-mediated tumor rejection following immunotherapy. A meta-analysis of three RCTs administering anti-PD-1 monotherapy or in combination with anti-VEGF to patients with advanced HCC (CheckMate-459,^7^ IMbrave150,^10^ and KEYNOTE-240^8^) showed that patients with HBV- or HCV-related HCC benefitted from ICI, whereas patients with HCC of a non-viral etiology did not.^25^ Non-viral etiology was associated with shorter survival in patients with HCC undergoing anti-PD-1 therapy, even after correction for potential confounding factors relevant for prognosis, such as severity of liver damage, macrovascular tumor invasion, extrahepatic metastases, performance status, and alpha-fetoprotein.^25^ MAFLD-HCC appears to be characterized by a particular TiME composition, including aberrant T cell activation, leading to tissue damage and impaired immune surveillance.^25^

Based on data from the IMbrave150 study, corroborated by post-registration evidence, between 19% and 22% of patients treated with atezolizumab plus bevacizumab experienced early disease progression following treatment.^26^,^27^

In this continuously evolving setting, the identification of mechanisms of resistance to ICI-based treatments is crucial to allow the identification of subgroups of patients that could experience the greatest benefit from different therapeutic regimens and, ultimately, to improve patient outcomes. In this review, we discuss the mechanisms of resistance to immunotherapies in HCC and review the main intrinsic and extrinsic mechanisms that might be exploited for therapy.

## Defining Therapeutic Resistance

Resistance to immunotherapy can be occurring in patients who are primary non-responders, or it can be acquired if it occurs after a period of documented response to therapy.^28^ The mechanisms proposed to explain the primary resistance to immunotherapy concern the reduced immunogenicity of the tumor, which can manifest itself as low expression of neoantigens, altered presentation of antigens and expression of immune co-inhibitory signals.^29^ HCC is generally considered a malignancy with low tumor mutational burden (TMB).^30^ As a consequence, the small proportion of somatic mutations leads to a lower expression of putative neoantigens. Antigen presentation in HCC is also negatively influenced by the expression of several co-inhibitory receptors that further suppress the function of T cell immunity responsible for tumor control.^31^

Our current understanding of acquired resistance is linked to the clonal evolution of the tumor towards a low immunogenicity phenotype after the initiation of immunotherapy.^28^ As with primary resistance, impaired functioning of the antigen presentation system plays a highly relevant role.^32^,^33^ Little evidence is applicable to HCC to date, where acquired resistance represents a field of active research.^34^

**Figure 1:**
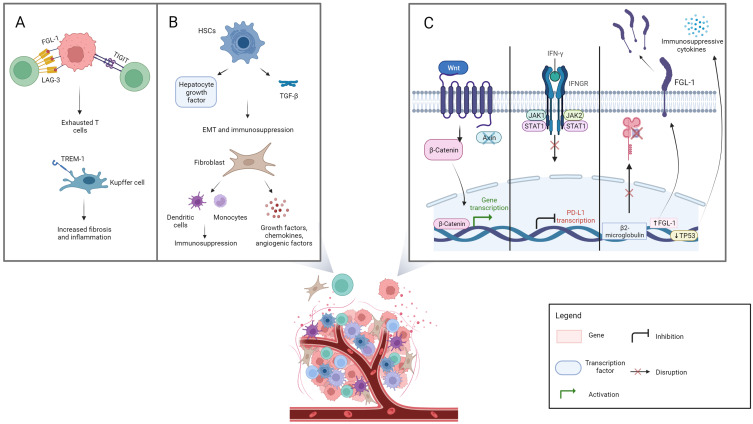
Selected mechanisms of resistance: extrinsic and intrinsic mechanisms are shown in panel **A** and **B**, and panel **C** respectively. Panel **A** represents the processes involved in T cell exhaustion, which are mediated by lymphocyte activation gene-3 and fibrinogen-like protein 1 (FGL-1) interaction, T cell immunoglobulin and immunoreceptor tyrosine-based inhibitory motif domain expression, and the development of inflammation led by Kupffer cells. Panel **B** shows the role of activated hepatic stellate cells and fibroblasts in immunosuppression. Panel **C** depicts intrinsic mechanisms of resistance (from left to right): the Wnt/beta-catenin pathway, interferon γ signaling with Janus kinase / signal transducer and activator of transcription pathway activation, and FGL-1 over-expression with beta-2-microglobulin dysfunction and p53 tumor suppressor gene-mediated immunosuppression. Created with BioRender.com.

**Abbreviations**: LAG-3, lymphocyte activation gene-3; FGL-1, fibrinogen-like protein 1; TIGIT, T cell immunoglobulin and immunoreceptor tyrosine-based inhibitory motif domain; TREM-1, triggering receptor expressed on myeloid cell-1; HSCs, hepatic stellate cells; TGF-β, transforming growth factor beta; EMT, epithelial-to-mesenchymal transition; β-Catenin, beta catenin; INF-γ, interferon γ; INFGR, interferon γ receptor; JAK, Janus kinase; STAT, signal transducer and activator of transcription; PD-L1, programmed cell death ligand-1; β2-microglobulin, beta-2-microglobulin; TP53, p53 tumor suppressor gene.

Another important conceptual characterization of resistance relies on its distinction between intrinsic, ie, linked to mechanisms that cancer cells implement to avoid immune rejection, and extrinsic, therefore external to tumor cells and linked to the characteristics of the TiME.^28^ In the clinic, it is accepted that these mechanisms often overlap in contributing to treatment failure. Figure 1 shows selected mechanisms of intrinsic and extrinsic resistance.

## Tumor-Intrinsic Mechanisms of Resistance

Among the many intrinsic mechanisms exploited by cancer cells to develop resistance to ICIs, oncogenic alterations have a primary role. Dysregulation of the Wnt/beta-catenin and Janus kinase (JAK) 1/2 pathways are among the key mechanisms studied to date, alongside the dysregulation of intracellular signaling in tumor cells and alteration of immune recognition processes.

### The Wnt/Beta-Catenin Pathway

Dysregulated Wnt/beta-catenin signaling is one of the major oncogenic alterations in HCC.^35^,^36^ Detailed genomic analysis has elicited that gain-of-function mutations in the CTNNB1 gene, which encodes beta-catenin, and loss-of-function mutations in AXIN1 occur in around 15–35% of HCC patients.^34^,^36^ As a result of these genetic changes, Wnt/beta-catenin signaling has been linked to progression, stemness, metastasis, and resistance in HCC patients.^34^,^36^,^37^ Studies have revealed that the pathway is characteristic of tumors that are enriched in cancer stem cells.^38^,^39^ Fan et al provided preclinical evidence on the effect of protein tyrosine kinase 2 hypomethylation enhancing HCC stemness via Wnt activation, ultimately causing resistance and increasing the risk of recurrence (p<0.01).^38^ Aberrant Wnt/beta-catenin activation has been associated with resistance to sorafenib and regorafenib.^36^,^40^ Harding et al demonstrated lower disease control rates (8.3% vs 40.2%) and progression-free (1.9 vs 5.3 months) and overall survival (2.0 vs 7.4 months) rates in HCC patients associated with over-activation of the Wnt/beta-catenin pathways.^40^ These poorer prognostic results were significantly associated with increased resistance rates (p<0.01).^40^ Similar findings have been corroborated by de Galarreta et al, who described that in a genetically engineered mouse model of HCC, the induced expression of exogenous MYC; Trp53-/- antigens causes immune evasion via upregulation of beta-catenin pathways, driving resistance to nivolumab and pembrolizumab.^38^ Incorporating next generation sequencing to identify patients with overactivated Wnt/beta-catenin pathways has the potential to identify HCC patients who would benefit from genome-targeted therapies. However, further studies into biomarker use are required before this can be integrated into standard clinical practice.

### P53 Tumor Suppressor Gene

TP53 is frequently defective in human cancers, including HCC, and its mutations can be caused by both viruses and chemicals.^41^ TP53 contributes to immune surveillance by recruiting CD8+ and T-helper cells, and its perturbations could promote an immunosuppressive environment.^42^,^43^ A recent study that analyzed 240 samples of HCC showed an increased rate of TP53 mutations in a group of tumors defined as “intermediate class”, characterized by a decreased immune infiltration and an immunosuppressive microenvironment. In this setting, TP53 demonstrates a critical role for immune evasion in HCC.^23^

### Interferon-γ Signaling Pathway

Interferon γ (INF-γ) is a cytokine secreted by T cells after the recognition of tumor neoantigens that act as a key regulator of immune response against tumor cells. The binding of INF-γ to its receptor on cancer cells induces the activation of the JAK-signal transducer and activator of transcription (STAT) signaling pathway, thus resulting in the subsequent transcriptional activation of PD-L1. This leads to an increased expression of PD-L1 on the tumor cells’ surface and subsequently, to the negative regulation of antitumor immunity.^44^ However, tumor intrinsic alterations of the INF-γ signaling pathway reduce the expression of PD-L1, thereby promoting resistance to ICI.^45^

Accordingly, mutations in genes involved in the INF-γ pathway, including loss of signaling-related genes (JAK 1/2) and amplification of suppressor genes, have been identified in patients that do not respond to anti-CTLA-4 and anti-PD-1 therapies, thereby reinforcing the suggested role of INF-γ signaling disruption in ICI resistance.^46^,^47^ Moreover, studies conducted on HCC tissues identified the presence of methylations in the suppressors of cytokine signaling genes SOCS-1 and SOCS-3, which are negative regulators of the JAK2-STAT signaling pathway. This epigenetic modification silences SOCS-3, thus leading to the constitutive activation of STAT3 in hepatoma cells. STAT3 is a transcription factor involved in the regulation of many cancer mechanisms, including resistance to apoptosis and treatment.^48^,^49^

### Tumor Mutational Burden

Somatic mutations occurring within neoplastic tissue may be relevant to cancer progression beyond their functional role in the protein they encode. Changes in the protein conformation induced by somatic mutations can elicit immune responses, through the unmasking of neoantigens. The positive correlation between somatic mutations and neoantigens entails that the higher the TMB of a tumor is, the greater its capacity would be to trigger a more efficient and diverse T cell response.^50^ Several studies have attempted to investigate TMB as a biomarker for ICI response.^51–53^ Although TMB alone is not sufficient to predict outcome, higher TMB scores have been associated with higher ICI efficacy in melanoma and non-small-cell lung cancer (NSCLC).^51^,^52^,^54^ TMB in HCC is relatively low compared to other solid tumors.^52^ Etiologic factors specific for HCC, such as virally induced chronic liver disease, appear to be related to a higher TMB, leading to a better response to ICI.^55^ In contrast, tumors arising in the context of MAFLD show a trend toward lower TMB.^56^ While retrospective evidence shows that higher TMB levels positively correlate with an improved 1-year OS compared to a lower TMB, the provision of immunotherapy is not routinely informed by TMB quantification in the clinic. Lack of a universally validated cutoff to define TMB-high, as well as difficulties in harmonizing sequencing platforms and depth are among the hurdles identified in the clinical applicability of this putative biomarker in HCC.^57^

### Dysfunction of the Antigen Presentation Machinery

The cytotoxic capacity of the host immune system against tumor cells is mediated by the effective presentation of cancer antigens and neo-antigens to CD8+ T cells. The latter is facilitated by major histocompatibility complex (MHC) proteins, which interact with T cell receptors (TCRs) on T lymphocytes and elicit cytotoxic activity.^58^ Downregulation of MHC proteins is related to immune escape of the tumor and could lead to ICI resistance.^59^ Mutations in beta-2-microglobulin, a gene associated with MHC stability on the cell surface, have been linked to ICI resistance in solid neoplasms.^60^ Likewise, the suppression of MHC class I proteins caused by methylation of its transactivator caspase recruitment domain 5 has been related to poor antigen presentation and reduced survival.^61^

### Fibrinogen-Like Protein 1

Fibrinogen-like protein 1 (FGL-1) is a hepatocyte protein^62^ involved in liver regeneration and cell proliferation.^63^ It is not only present in the liver, but it also exists in plasma, where it, however, has not been implicated in coagulation.^62^ FGL-1 has been demonstrated as an important ligand for lymphocyte activation gene-3 (LAG-3 or CD223), a transmembrane protein that, like PD-1, represents one of several immune checkpoints considered gatekeepers of immune responses.^64^ The interaction between the LAG-3 extracellular domain and the FGL-1 domain is independent from the antigen presenting machinery; their binding is less effective in resting T cells that express minimal LAG-3 levels.^65^,^66^ Impediment of the FGL1-LAG-3 interaction in mouse models potentiates anti-tumor immunity.^65^ Using this interaction as a therapy target could allow to avoid the resistance mechanisms caused by the alteration of antigen presentation mechanisms, considering that the latter would not be directly involved. Moreover, FGL-1 expression within the tumor appeared to be correlated with the progression and prognosis of HCC, suggesting that FGL-1 could be used not only as a molecular target but also as a potential prognostic biomarker.^67^ More research is needed in order to understand if LAG-3 through FGL-1 blockade could have a role as a new therapeutic strategy to overcome resistance to immunotherapy. In addition to its therapeutic role, it will be important to analyze the prognostic potential of FGL as a prognostic marker. In fact, FGL-1 expression in circulating tumor cells has been related to a poor prognosis in patients with HCC who underwent resection.^68^

## Therapy-Associated Mechanisms of Resistance

### Antidrug Antibodies

The widespread use of immunomodulatory agents has drawn attention to their potential immunogenicity, as well as their related effects on the safety and efficacy of treatment.^69^ While the majority of monoclonal antibodies is able to induce antidrug antibody (ADA) formation, following interaction with the host immunity,^70^ the risk of ADA development is intrinsically drug-specific, with diverse ranges of ADAs being reported across studies. Treatment with B-cell depleting agents appears associated with a lower risk of immunogenicity, compared to agents that enhance T cells functions.^69^ Depending on the protein composition of the monoclonal antibody, a “vaccine-like” reaction may develop with first the generation of low-affinity IgM antibodies, followed by the appearance of high-affinity IgG antibodies; in the presence of low immunogenic monoclonal antibodies, the ADAs will mostly only be responsible of a minimal clinical effect.^71^ Each patient may have non-neutralizing or neutralizing ADAs,^72^ capable of reducing the efficacy of monoclonal antibodies by altering their bioavailability and/or accelerating their clearance from circulation.^73^ For most agents, the titers and percentages of ADA-positive patients are reported, but the clinical consequences of ADAs are not investigated.^70^ A recent observational study examining 132 patients with advanced HCC treated with atezolizumab plus bevacizumab, showed that the emergence of high titers of ADA (>1000 ng/mL) as early as 3 weeks post commencement of systemic therapy, was associated with shorter OS, even after adjusting for various confounding factors. Patients with higher ADA levels exhibited reduced systemic exposure to atezolizumab, as well as impaired proliferation and activation of peripheral CD8+ T cells.^74^ Treatment-related anti-tremelimumab and anti-durvalumab antibodies have also been reported in the HIMALYA trial. Different antibody percentages have been identified, depending on whether the treatment involves the combination of the two drugs or the single agent: anti-durvalumab antibodies were detected in 3.1%, 2.5%, and 4.6% of patients receiving a single dose of 300 mg tremelimumab plus 1500 mg of durvalumab every 4 weeks (STRIDE regimen), durvalumab as single agent, and 75 mg of tremelimumab every 4 weeks for four doses plus 1500 mg of durvalumab every 4 weeks (T751D), respectively; neutralizing antibodies were detected in 1.7%, 0.7%, and 0% of patients, respectively. Anti-tremelimumab antibodies were detected in 11.0% patients receiving the STRIDE regimen and 21.6% receiving T751D at any visit; neutralizing antibodies were detected in 4.4% and 15.7% patients, respectively.^11^ Relationship with clinical outcomes was not investigated. Considering the effectiveness of immunotherapy and its first-line indication in advanced HCC, further studies are needed to investigate prevalence and clinical significance of antidrug antibodies and their consequences on therapy efficacy. A call for the universal evaluation of ADAs across clinical trials as a mechanism of resistance is needed to ensure a better and more complete understanding of their significance in the delivery of immunotherapy.

## Tumor Extrinsic Mechanisms: The Immune Contexture of the Tumor Microenvironment

In HCC, the TiME is known to play a critical role in HCC development and progression. It consists of a dynamic network of growth factors and inflammatory cytokines, extracellular matrix proteins, endothelial cells, lymphocytes, TAMs, hepatic stellate cells (HSCs), and stromal cells, including cancer-associated fibroblasts (CAFs).^75^,^76^ The TiME in HCC exhibits a high functional heterogeneity, where certain immune infiltrating cells can either promote a favorable antitumor immune response or limit effective immune surveillance.^77^ With often functionally opposing roles, infiltrating cells composing the TiME constitute a neoplastic niche, where the tumor can proliferate rapidly, escape from the host’s defense systems that are directed against transformed cells, and facilitate the invasive behavior of cancer cells.^76^ This bidirectional effect of the TiME in influencing the biological characteristics of the tumor has been termed immunoediting. It consists in a dynamic process, where the immune system not only protects against cancer cell growth but is also able to promote tumor progression, either by establishing a facilitating TiME or by selecting tumor cells that are more fit to survive.^78^ Immunoediting is traditionally divided into three phases: elimination, where transformed cells are destroyed by a competent immune system; equilibrium, where cells that managed to survive the immune attack start the editing process; and escape, the last phase, where the tumor grows progressively, becomes clinically apparent and establishes an immunosuppressive TiME.^78^ This process is not only involved in tumor development, but it can also occur in a patient undergoing immunotherapy.^79^ Understanding the interactions among the immune response, oncogenic signaling, and the TiME is now more than ever critical to improve the efficacy of immunotherapies, which are highly reliant on T cell immune reconstitution.^22^,^80^

A more in-depth view of the TiME organization could prove useful. A recent study by Sheng et al^81^ focused on the spatial characteristics of the TiME in HCC. Using the imaging mass cytometry, regional function units have been identified, each one of them with a distinct distribution of stromal and immune cells. The presence of specific regions enriched with cancer cells has been associated with worse patient OS and PFS. At the same time, a poor prognosis was related to the enrichment of Kupffer cells, which negatively affected the activation of T cells.^81^ Indeed, technologies able to return data on spatial organization of the TiME could prove able to discover novel biomarkers and therapeutic targets.^82^ Having more information on the constitution of the TME, it would be possible to act on its remodeling, which could be one of the important immunotherapeutic approaches to improve HCC treatment.^83^

### Lymphocyte Activation Gene-3

LAG-3 is a type I transmembrane protein with four extracellular Ig-like domains that is selectively transcribed into activated NK and T lymphocytes.^84^ It has structural similarities with another MHC class II ligand (MHC-II), called CD4, with which it shares approximately 20% amino acid sequence identity.^85^ While the interaction between MHC-II and CD4 supports T helper cells activation, the overexpression of LAG-3 downregulates antigen-dependent T helper (CD4+) responses in vitro.^84^ LAG-3 is not instrumental in the induction phase of the immune response due to its engagement only after lymphocyte activation.^86^ Its expression can be induced by infective^87–90^ and tumor-related antigens, and its overexpression leads to the generation of exhausted T cells, which ultimately results in immune escape.^91–96^ Exhausted CD4+ and CD8+ tumor-infiltrating T cells are defective in cytokine production.^97^ LAG-3 blockade has been demonstrated to reinvigorate exhausted T cells and strengthen anti-infection immunity, although to a lesser extent, compared with that caused by PD-1 blockade.^98^ Preclinical studies have suggested that the co-engagement of LAG-3 and PD-1 further suppresses interleukin-2 (IL-2) production, which is directly involved in T lymphocyte differentiation, arguing that LAG-3 and PD-1 act synergistically to restrain the antigenic stimulation of CD4+ T cells.^99–101^ Based on these assumptions, several clinical trials involving patients with different solid neoplasms, evaluating both the safety of LAG-3 inhibitors and the expression of LAG-3 as an outcome marker, have been concluded or are currently recruiting (clinicaltrials.gov).

### T Cell Immunoglobulin and Immunoreceptor Tyrosine-Based Inhibitory Motif Domain (TIGIT)

TIGIT is another immune checkpoint molecule first described in 2009, able to exert its immunosuppressive effects by binding to the poliovirus receptor (PVR or CD155), which is highly expressed in various neoplasms, and to PVR-like protein.^102^ TIGIT is also able to modulate cytokine production in dendritic cells.^102^ TIGIT is expressed on NK, NKT, CD8+, Treg, and memory CD4+T cells; in normal conditions, it protects normal cells from NK-mediated cytotoxicity and counter inhibits the NK-mediated killing of tumor cells.^103^ It has been hypothesized that the presence or the overexpression of TIGIT poses a metabolic barrier to T cell function^104^ and in preclinical studies, TIGIT blockade has been shown to enhance T cells responses.^105^,^106^ Overexpression of CD155 in HCC may lead to a defective immune response by upregulating TIGIT.^107^ Given these premises, it is understandable why TIGIT can be used as a therapeutic target together with other immune checkpoints, particularly in combination with PD-1 blockade: the simultaneous blockade of TIGIT and PD-1 enhances CD8+ T cell function in HCC.^108^,^109^ Both preclinical and clinical trials are actively investigating anti-TIGIT antibodies.^104^

### CD8+ T Cell Infiltration and Functional Phenotype

CD8+ cytotoxic T cells normally play a crucial role in anti-tumor immune response. Continuous exposure to antigens, such as during chronic infections and cancer, leads to failure of efficient CD8+ T cell development and, ultimately, their exhaustion.^107^,^110^ Exhausted CD8+ T cells have both a limited effector function, caused by the loss of IL-2, tumor necrosis factor-α and interferon-γ production, as well as a high co-expression of inhibitory receptors, such as PD-1, CTLA-4, TIGIT, and LAG-3, together with extensive transcriptional changes, compared to effector or memory CD8+ T cells.^111^ As a result, exhausted CD8+ T cells fail to control tumor growth, but implementing effective methods to reinvigorate them can significantly impact cancer progression,^112^ such as with ICI. HCC more often presents with an exhausted phenotype of CD8+ T cells in the TiME, compared to effector CD8+ T cells. In particular, an increase in the number of exhausted cells has been observed in advanced stages, compared to early stages.^113^ Moreover, the presence of exhausted CD8+ T cells expressing high levels of PD-1 has been described as increased in parallel with tumor stages, suggesting that the severity of CD8+ T cell exhaustion is related to HCC progression.^114^ In accordance with what has already been said, HCC patients with high proportions of T cells expressing PD-1 showed significantly dismal postoperative survival after hepatectomy and a high risk of recurrence.^114^ In addition to the degree of PD-1 expression, the ratio between effector T cells and exhausted CD8+ T cells proved to be associated with disease control in anti-PD-1-treated patients.^115^ Recruiting more active T cell populations could allow for a greater response to ICI.

### The Triggering Receptor Expressed on Myeloid Cell-1

Triggering receptors expressed on myeloid cells (TREMs) are a family of cell-surface receptors expressed broadly on myeloid cells, capable of stimulating neutrophil- and monocyte-mediated inflammatory responses.^116^,^117^ TREM-1 was first described as up-regulated in infections^117–119^ and inflammatory conditions,^120–122^ but also in autoimmune diseases.^123^ It was later described that cancer cells in NSCLC can directly up-regulate TREM-1 expression in the patients’ macrophages, which is associated with cancer recurrence and poor survival.^124^ In liver fibrosis, TREM-1 expression appeared robustly increased in Kupffer cells and monocytes/macrophages, suggesting a role in the inflammatory liver response and in fibrotic liver disease.^125^ The inhibition of the TREM-1 signaling pathway in a preclinical model proved to regulate the inflammatory response and attenuate the development of inflammation-associated HCC.^126^ Furthermore, in HCC, TREM-1 expression on HSCs was related to the aggressive tumor behaviour.^127^ Knowing the role of TREM-1 in activating inflammation and promoting carcinogenesis, this receptor could be studied not only as a future target for immunotherapy but also as a potential biomarker.^128^

### Transforming Growth Factor Beta

Transforming growth factor beta (TGF-beta), mainly secreted by HSCs, plays an important role in liver carcinogenesis exerting proinflammatory/fibrotic, tumor suppressive and/or pro-metastatic effects.^129^ In the early stages of tumor development, high levels of TGF-beta have been associated with better prognosis, whereas increased invasiveness and dedifferentiation have been reported in advanced tumors. These effects suggest that in early stages TGF-beta exerts its tumor suppressor function, but following disease progression it contributes to the development of a malignant phenotype.^129^ Studies have reported that TGF-beta may induce a decrease of E-cadherin, characteristic of epithelial cells, and an up-regulation of mesenchymal genes, thus leading to an epithelial-to-mesenchymal transition.^130^

Moreover, it is known that TiME-derived TGF-beta is involved in the suppression of antitumor T cell response. In fact, an increase in TGF-beta signaling through SMAD3 transcriptional regulation may cause the upregulation of PD-1 expression, thus inducing T cell exhaustion and conferring resistance to PD-1 blockade therapies.^131^

### Activated Hepatic Stellate Cells

HSCs are mesenchymal cells located in the space of Disse between hepatocytes and sinusoidal endothelial cells.^132^ They represent the principal source of extracellular matrix formation^132^ and their presence is vital to hepatocellular function, as well as the liver’s response to injury.^133^ The exposure to any noxious stimulus, such as viral infection or alcohol, causes HSC activation and increased deposition of the extracellular matrix, ultimately leading to fibrinogenesis.^134^ Scar tissue formation is aimed at protecting the liver from further damage; however, sustained activation of HSCs leads to chronic fibrosis and cirrhosis.^135^,^136^ HSCs are an important source of growth factors in the liver, but they are also sensitive to these factors.^136^ In HCC, HSCs can promote chemoresistance through the secretion of hepatocyte growth factor, which provokes upregulation of the cancer stem cell-like characteristics of epithelial tumor cells and epithelial-to-mesenchymal transition.^137^,^138^ The activation of HSCs also contributes to immunosuppression: they are able to convert mature peripheral blood monocytes into myeloid-derived suppressor cells,^139^,^140^ they can induce T cell apoptosis through PD-L1 expression,^141^ as well as Treg cell accumulation inside the liver.^142^ Knowing their role in the regulation of immune response, targeting these cells may improve immunotherapy efficacy.

### Cancer-Associated Fibroblasts

Cancer-associated fibroblasts (CAFs) represent a constitutive group of stromal cells of the TiME, capable of promoting hepatocarcinogenesis.^143^,^144^ While their origin is not yet fully understood, and a unique marker for their identification is missing,^144^ it appears clear that these cells can produce several factors, such as growth factors, chemokines, and angiogenic factors that support tumor-cell proliferation and invasion.^145–148^ Additionally, CAFs appear to sustain a crosstalk with the TiME, recruiting dendritic cells and monocytes responsible for immune response suppression,^149–152^ and they also have the ability to increase PD-1 expression.^153^ Indeed, CAFs infiltration in the TiME is associated with a response to immunotherapy.^154^ Moreover, their interaction with macrophages promotes the construction of a tumor immune barrier, which is associated with the efficacy of ICI.^155^ Their role in immunosuppression justifies the identification of these cells as a therapeutic target. Both preclinical and clinical studies of CAF-targeted therapies are under investigation,^156^,^157^ but more experience in HCC is needed.

### Influencing the TiME: The Gut Microbiome

The gut microbiome is a complex entity that comprises different microorganisms capable of performing immunomodulatory activities.^158^ The bacteria and their metabolites are constantly interacting with immune cells at the gut mucosa to coordinate immune responses in the absence of inflammation.^159^ This so-called “homeostatic immunity” is controlled by several factors, including antibody responses to commensal bacteria, innate-like T cell responses, as well as the microbiota and its metabolites. Given the role of the gut microbiome in the immune system, it is unsurprising that it has been shown to modulate T cell infiltration of solid tumors and efficacy of ICIs.^160^ Species, such as Bifidobacterium^161^ and A. Muciniphila^160^,^162^ in particular, have been the focus of extensive research. Antibiotics are well documented as having a sustained impact on gut microbiota composition,^163^,^164^ with proton-pump inhibitors^165^,^166^ and corticosteroids^167^ also likely to modulate the gut microbiome. There have been a number of association studies investigating the impact of these medications given prior to or concomitantly with ICIs. Concomitant use of these three drug types has been associated with poor patient outcomes in retrospective analyses,^168^,^169^ with some evidence that they have a cumulative effect on predicting OS and PFS in patients with advanced cancer receiving ICI therapy.^170^ It has also been shown that use of antibiotics during the 30 days prior to the start of ICI therapy significantly reduces the likelihood of treatment response and OS (independent of confounding factors).^171^ In HCC, use of antibiotics within 30 days before or after systemic therapy initiation was associated with a worse OS and PFS, not only in ICI recipients but also in patients treated with anti-VEGF, TKI, and placebo. These results highlight the complexity of HCC composition, its interaction with underlying hepatopathy, and the pleiotropic effects of molecular therapies for HCC.^172^ It is essential to establish whether this is a causative relationship mediated by changes in the gut microbiome,^173^ as the use of concomitant or prior medications provide a potential modifiable factor in response to ICIs.

## Conclusions

ICIs have revolutionized the treatment of advanced hepatocellular carcinoma. Nevertheless, resistance to immunotherapy remains a major obstacle in the treatment of HCC. Both intrinsic and extrinsic mechanisms contribute to the development of resistance. Among the intrinsic mechanisms, TMB, which refers to the total number of somatic mutations in the tumor, plays a major role in the regulation of the immune response. Notably, a high TMB, which results in a higher presence of neoantigens, has been linked to a greater response to treatment, even if its role has not been prospectively evaluated in ICI.^174^ Tumor intrinsic mutations can occur in genes involved in the regulation of immune response. The overactivation of beta-catenin is related to lower CD8+ T cell infiltration and PD-L1 expression, thus resulting in resistance to ICIs.^175^ Moreover, inactivating mutations of TP53, an oncosuppressor gene, have been linked to tumor progression and resistance to ICIs.^23^,^43^ It has also been found that dysregulation of genes involved in the INF-γ signaling pathway, as well as defects in antigen presentation mediated by the MHC machinery, may contribute to ICI resistance.^45^,^59^ Cancer cells overexpress FGL-1, which interacts with LAG-3 on T cells, leading to T cell exhaustion and subsequently, repression of the antitumor response.^65^

T cell exhaustion belongs to the tumor extrinsic mechanisms of resistance, for which TiME is responsible. The suppression of T cell function may be exerted by the overactivation of TIGIT, which is caused by the overexpression of its ligand, CD155, thus creating an immunosuppressive microenvironment and inducing tumor escape.^104^ Additionally, studies show that increased expression of TREM-1 elicits T cell dysfunction through the modulation of PD-L1, which impairment fails to improve the related immunosuppression.^176^ Additionally, HSCs^141^,^142^ and CAFs^152^ can also contribute to the development of an immunosuppressive framework. The gut microbiome’s influence on the TiME has been widely acknowledged.^160^

To expand the spectrum of cancer patients that could benefit from immunotherapy, more targets and innovative approaches need to be considered in future clinical trials.^66^ A number of strategies have been proposed to overcome resistance to immunotherapy. In addition to using triplets ICI-regimens, also other mechanisms are being explored: among them, reinvigorating exhausted T cell response seems to be one of the most promising, with pre-clinical and clinical studies currently investigating the blockade of other immunosuppressive promoters, such as LAG-3 and TIGIT (clinicaltrials.gov). Moreover, the modification of the immunosuppressive drift of the TiME could improve ICI-efficacy. Many mechanisms are thought to be responsible for the creation of an immunosuppressed niche for the tumor, but our comprehension of these complex interactions remains incomplete.

Another proposed method to overcome resistance to immunotherapy are vaccines. Tumor vaccines are intended to facilitate tumor-specific immune responses by targeting tumor antigens.^177^ At the present time, the technologies investigated for vaccines in HCC so far include dendritic cell vaccines and peptide vaccines, such as AFP and glypican-3 (GPC3).^178^ All these strategies have been demonstrated to be safe and although monotherapy with vaccines showed limited clinical efficacy, this could be attributed to the diverse features of HCC tumors and vaccine design, or even a combination of both.^179^ The selection of the right target for vaccines is crucial. The risk of toxicity could be magnified by the expression of the vaccine target in non-malignant tissues, such as in the case of tumor-associated antigens, which are not specifically present only in cancer cells.^180^ Furthermore, the development of tolerance mechanisms is considered a possible issue.^180^ A new technology for vaccines could represent a future therapeutic strategy: neoantigens are new protein sequences specific for each patient, resulting from mutations appearing in tumor cells.^181^ Neoantigens are highly tumor-specific, as they are not present in normal cells, and they possess great immunogenic capacity, but they require great MHC affinity to be processed by APC and to then be recognized by T cells.^179^ The combination of neoantigen-based vaccines with ICIs has been under investigation in several solid tumors, like NSCLC, bladder cancer, and melanoma.^182^,^183^ The encouraging results from previous studies paved the way for the application in HCC: currently clinical trials involving neoantigen-based vaccines in combination with anti-PD-1 (NCT04251117, NCT05269381, and NCT04912765) or anti-PD-1 plus anti-CTLA-4 immunotherapy (NCT04248569) in HCC are recruiting, both in Phase 1 and Phase 2.

Additionally, chimeric antigen receptor (CAR) T cell therapy targeting HCC antigens may also be a promising strategy to overcome ICI resistance. CARs are engineered, non-MHC-restricted receptors that redirect the specificity, function, and metabolism of T cells, ultimately leading to an improved T cell function.^184^,^185^ They possess an extracellular region of a receptor that is able to recognize a specific antigen and, consequently, lead to cell death.^186^ CAR-T cell therapy has already shown promising results in some oncohematological settings,^187^ while its role in solid tumors still requires further research. Among the major obstacles that remain to be overcome concern the tumor mass infiltration and the presence of a particular immunosuppressive TiME.^188^ The ability of CAR-T to reach cancer cells in solid tumors is invalidated by physical barriers belonging both to the tumor itself, for instance, the stromal tumor structure, and to the CAR-T mobility.^189^ Furthermore, the lack of a single target tumor reduces the effectiveness of this treatment and increases the risk of toxicity on non-malignant cells.^188^,^189^ In addition, even after the identification of an appropriate single target, the tumor could promote an antigen escape, downregulating the target antigen expression.^189^ Combining multiple antigens is an investigated strategy.^190^ For HCC, the tumor-specific GPC3 antigen has been identified as a target for CAR-T cell therapy.^191–193^ Several clinical trials for advanced HCC patients are investigating GPC3 CAR-T cells (clinicaltrails.gov). The combination of CAR-T cells and ICIs could represent a future therapeutic scheme capable of, on the one hand, stimulating the immune system, and, on the other hand, effectively hitting the chosen tumor targets. However, the problem of identifying a tumor marker of HCC that allows a more targeted therapy remains open, as well as the identification of a marker that allows to predict the response to immunotherapy. Various biomarkers have already been proposed, but none of these have been validated in HCC yet, and further research is needed to determine their clinical utility.

Much remains to be understood about the mechanisms of resistance to immunotherapy; further translational studies are required to identify the patients who will respond most to this type of therapy and to avoid unnecessary side effects in patients who will not respond to immunotherapy. More research is needed to develop effective strategies to overcome immunotherapy resistance.
